# Mapping Powdery Mildew (*Blumeria graminis* f. sp. *tritici*) Resistance in Wild and Cultivated Tetraploid Wheats

**DOI:** 10.3390/ijms21217910

**Published:** 2020-10-24

**Authors:** Rosanna Simeone, Luciana Piarulli, Domenica Nigro, Massimo Antonio Signorile, Emanuela Blanco, Giacomo Mangini, Antonio Blanco

**Affiliations:** 1 Genetics and Plant Breeding Section, Department of Soil, Plant and Food Sciences (DiSSPA), University of Bari Aldo Moro, Via Amendola 165/A, 70126 Bari, Italy; luciana.piarulli@libero.it (L.P.); domenica.nigro@uniba.it (D.N.); massimoantonio.signorile@uniba.it (M.A.S.); antonio.blanco@uniba.it (A.B.); 2 Institute of Biosciences and Bioresources, National Research Council, Via Amendola 165/A, 70126 Bari, Italy; emanuela.blanco@ibbr.cnr.it (E.B.); giacomo.mangini@ibbr.cnr.it (G.M.)

**Keywords:** *Blumeria graminis*, powdery mildew, wheat, disease resistance, association mapping, GWAS

## Abstract

Wheat is the most widely grown crop and represents the staple food for one third of the world’s population. Wheat is attacked by a large variety of pathogens and the use of resistant cultivars is an effective and environmentally safe strategy for controlling diseases and eliminating the use of fungicides. In this study, a collection of wild and cultivated tetraploid wheats (*Triticum turgidum)* were evaluated for seedling resistance (SR) and adult plant resistance (APR) to powdery mildew (*Blumeria graminis*) and genotyped with a 90K single nucleotide polymorphism (SNP) array to identify new sources of resistance genes. The genome-wide association mapping detected 18 quantitative trait loci (QTL) for APR and 8 QTL for SR, four of which were identical or at least closely linked to four QTL for APR. Thirteen candidate genes, containing nucleotide binding sites and leucine-rich repeats, were localized in the confidence intervals of the QTL-tagging SNPs. The marker IWB6155, associated to *QPm.mgb-1AS,* was located within the gene *TRITD1Av1G004560* coding for a disease resistance protein. While most of the identified QTL were described previously, five QTL for APR *(QPm.mgb-1AS, QPm.mgb-2BS, QPm.mgb-3BL.1, QPm.mgb-4BL, QPm.mgb-7BS.1)* and three QTL for SR (*QPm.mgb-3BL.3, QPm.mgb-5AL.2, QPm.mgb-7BS.2*) were mapped on chromosome regions where no resistance gene was reported before. The novel QTL/genes can contribute to enriching the resistance sources available to breeders.

## 1. Introduction

Wheat is the most widely grown crop in the world and provides nearly 55% of the carbohydrates and 20% of the daily protein consumed worldwide. It contributes remarkably to human nutrition as it represents the staple food for about one third of the world’s population [1]. With a predicted world population of 9 billion in 2050, demand for wheat grain is predicted to increase by 50–110% from today’s levels [2]. To meet this demand, innovative cropping systems and genetic improvement for wheat yield, tolerance to abiotic stresses, pathogens and pests, and nitrogen and phosphorous use efficiency, represent a priority for all countries [3].

Wheat is attacked by a large variety of pathogens, mostly of fungal origin. Powdery mildew (PM), caused by *Blumeria graminis* (DC) Speer f. sp. *tritici* Em. Marchal (syn. *Erysiphe graminis* f. sp. *tritici*) (*Btg*), is a disease of major importance, as foliar damage results in yield loss in many wheat growing areas with humid or semi-continental environments [4]. The use of resistant cultivars has proved to be an effective and environmentally safe strategy for controlling wheat pathogens and eliminating the use of fungicides [5,6]. However, natural populations of the pathogen consist of multiple races, and new ones continue to be formed as a result of genetic recombination that could lead to the breakdown of resistance genes. For example, the defeat of *Pm17, Pm3a, and Pm4a* was reported in some Eastern and mid-Atlantic regions of USA [7,8,9] and of *Pm8* in China [10]. Consequently, most resistance genes tend to become ineffective within a short period, and therefore new sources of resistance to new races are needed for a more effective and durable resistance [11]. The common management strategy has been to replace cultivars when their resistance is no longer effective, and the diversification of sources of resistance, provided that an adequate number of resistance genes are available [12].

Resistance to diseases in crops is typically classified in two main types: qualitative resistance, which is determined by major race-specific resistance genes, and quantitative resistance controlled by several genes (quantitative trait loci, QTL) with additive effects [13]. The race-specific resistance is attributed to the presence of a major resistance gene (*R* gene) and to a corresponding pathogen avirulence gene (*Avr* gene); the plant *R* gene codes for a receptor that is activated by a pathogen effector [14], and it usually confers resistance at all plant stages. So far, 67 genes for PM resistance (*Pm1-Pm67*) that map to all wheat chromosomes have been identified [15,16]. Some of these genes have been transferred from wild and domesticated related species (*T*. *turgidum*, *T. timopheevii, T. monococcum, T. spelta, Ae. tauschii, Ae. longissima, Ae. speltoides*, *Ae. ovata,* or from more distant species, such *Secale cereale* and *Dasypyrum villosum*) to cultivated durum and bread wheat [17]. Ten PM resistance genes, all encoding the nucleotide binding sites and leucine-rich repeat (NBS-LRR) proteins, have been cloned to date: *Pm3* [18], *Pm38* [19], *Pm8* [20], *Pm46* [21], *Pm2* [22], *Pm21* [23,24,25], *Pm17* [26], *Pm60* [27], *Pm5* [28], *Pm24* [29] and *Pm41* [30].

A well-studied PM susceptibility gene is MLO (Mildew-Locus-O) which was first demonstrated in barley in 1942 [31], and later reported in rice and wheat [32,33]. In barley, the recessive allele *mlo* (natural and induced loss-of-function mutations of *Mlo)* confers broad-spectrum resistance against most *Bgt* races and with long-standing efficacy [31,34]. Recently, *Mlo* mutants showing good *Bgt* resistance have been generated in bread wheat by different technologies [35,36].

A second type of resistance to powdery mildew is determined by two to several non-race specific genes with additive effects and commonly effective at adult-plant stage (often designated polygenic resistance, horizontal resistance, quantitative resistance, adult plant resistance, APR). Quantitative resistance is usually analyzed by quantitative genetics methods, including estimating genetic components, heritability, and effective gene numbers. So far, several studies have mapped more than 100 QTL on all chromosomes [17], none of which have yet been cloned. The classical genetic analysis of quantitative traits is carried out on segregating biparental populations evaluated for the traits of interest and genotyped with DNA-based molecular markers. The genetic dissection of a complex trait into discrete quantitative trait loci exploits the association between molecular markers and QTL [37]. This approach requires the cost- and time-consuming development of large mapping populations (usually recombinant inbred lines and doubled-haploid lines) segregating for the quantitative trait, and the linkage analysis is restricted to loci in genomic regions containing polymorphisms between the two parental lines. The resolution power is rather poor due to the low number of crossing-over and the strong linkage disequilibrium (LD), making further analysis necessary for fine mapping of the detected QTL region covering many cM [38].

The alternative approach to the traditional linkage-based QTL mapping is the Genome-Wide Association Study (GWAS) based on LD and that uses a variety of genotypes (germplasm accessions, landraces, breeding lines, cultivars) representing the products of hundreds of recombination cycles, thus giving a more precise location of individual QTL [39]. The major limitations of GWAS lie in the large frequency of false-positive associations resulting from population structure, relative kinship among individuals, and multiple testing of thousands of markers, and of false-negatives that result after application of Bonferroni or false discovery rate corrections [40]. Merits and demerits of GWAS in some major crops (maize, wheat, rice, sorghum and soybean), the correction required to address the limitations of GWAS, and the utilization for crop improvement were discussed by Gupta et al. [41].

In this paper, we report the assessment of the genetic diversity for seedling resistance (SR) and adult plant resistance (APR) to powdery mildew in a panel of wild and cultivated tetraploid wheats including seven subspecies of *Triticum turgidum* (*durum*, *turanicum*, *polonicum*, *turgidum*, *carthlicum*, *dicoccum* and *dicoccoides*) by exploiting genomic resources and single nucleotide polymorphism (SNP) markers. The primary objectives were to perform a GWAS to identify (a) new sources of PM resistance genes; (b) provide the precise map position of associate SNP markers on the high-density SNP-based consensus durum map [42]; (c) identify candidate genes. Each *T. turgidum* subspecies is easily crossable with both cultivated durum and common wheats and simply breeding procedures enable an efficient introgression of desirable alleles from each subspecies into the cultivated gene pool [43]. The identification of genetic loci controlling SR and APR to powdery mildew will provide additional genetic resources to breeders for improve commercial cultivars of durum and bread wheat, as well as the opportunity to develop tightly linked markers to be used in marker-assisted selection (MAS) programs.

## 2. Results

### 2.1. Powdery Mildew Resistance in the Tetraploid Wheat Collection.

The tetraploid wheat collection, including 221 accessions of wild and cultivated genotypes belonging to seven *T. turgidum* subspecies, was evaluated for APR in a greenhouse experiment. The PM susceptible control cv. Ciccio, repeated ten times in each replication, always showed high levels of infection type (IT) (7–8) by the pathogenic fungus. Mean values of APR for each examined accession are reported in Table S1, and means, standard deviations and ranges for the whole collection and for each *T. turgidum* subspecies are reported in Table 1. Phenotypic variation in the whole collection ranged from highly resistant genotypes (22.6% of the accessions with IT from 0 to 2) to highly susceptible genotypes (22.5% of the accessions with IT from 6 to 9). The percentage of resistant accessions within the examined subspecies was highly variable, with values ranging between 0% for ssp. *turanicum* to 8.0% for ssp. *durum*, 83.3% for ssp. *dicoccum* and 88.9% for ssp. *dicoccoides*.

The seedling response (Stakman scale 0–4) [44] to *Bgt* isolate O2 ranged from 0 to 4 in the whole collection, with 12.7% of highly resistant accessions (IT from 0 to 1.0) and 56.1% of highly susceptible accessions (IT = 4.0). Few accessions of cultivated durum wheat showed PM resistance (5 out of 125 genotypes), while a high percentage of ssp. *dicoccum* and ssp. *dicoccoides* accessions showed high resistance levels (55.6% and 88.9%, respectively). No resistant accession was found within the ssp. *turanicum* and ssp. *turgidum*.

**Figure 1:**
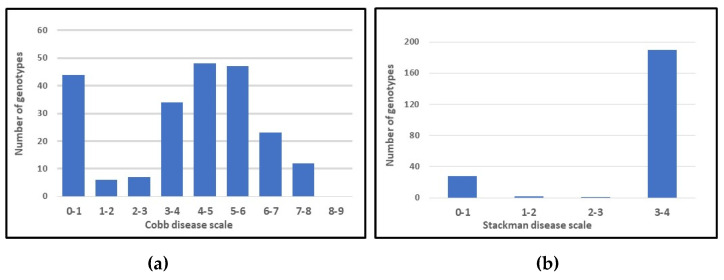
Frequency distribution of adult plant resistance **(a)** and seedling resistance **(b)** to powdery mildew in a collection of 221 tetraploid wheats.

The frequency distributions of infection type for APR and SR (Figure 1) were shifted toward low IT levels (PM resistance accessions), and the Shapiro–Wilks test [45] indicated significant deviations from normal distribution. The square root, arcsine and log transformation of IT values did not improve the normality of the original phenotypic data (data not shown). Overall, more examined accessions showed APR (22.6%) than seedling resistance (13.6%), suggesting that some genes for APR might be different from those expressed at seedling stage. Twenty-six out of 221 accessions (11.8%), including nine genotypes of ssp. *dicoccum* and eight genotypes of ssp. *dicoccoides*, showed a combined resistance at seedling and adult plant stages (Table 1 and Table S1), and can be considered a good source of PM resistance to be used in wheat breeding programs.

### 2.2. Association Analysis for Bgt Resistance

The tetraploid wheat collection was genotyped by the Illumina 90K iSelect array containing 81,587 SNPs [46]. After removing failed and monomorphic markers, SNPs with more than 20% missing data or with a minor allele frequency of less than 5.0%, and unmapped markers, a total of 19.393 SNPs mapped in the durum consensus map [42] were retained for GWAS. The total length of the genetic map was 2554.8 cM with an average distance of 7.6 cM between adjacent markers (Table S2). A total of 8377 markers localized on the genome A and 11,002 on the genome B, with a total length of 1278.8 and 1276.2 cM, respectively. The lengths of individual chromosomes varied from 131.2 cM (chromosome 6A) to 217.0 cM (chromosome 5A), while the number of markers ranged from 912 (4A) to 2057 (2B). The SNP density varied from a minimum of 5.2 for chromosomes 4A e 5A to a maximum of 11.6 for chromosome 1B.

In order to consider possible confounding effects of population structure and relative kinship, and then to minimize false-positive associations, data for APR and SR were preliminarily analyzed by four statistical models (GLM, GLM+Q, MLM+K, MLM+K+Q), taking into account the Q matrix and the Kinship (K) matrix. The inspection of Q–Q plots (Figure S1) indicated significant deviations of observed -log_10_(*p*) values from the expected -log_10_(*p*) distributions for the GLM and GLM+Q models and a closer observed and expected distributions for the MLM+K and MLM+K+Q models. The MLM+K+Q model was definitively used for the GWAS analysis as it produced the best results. However, the MLM+K+Q for the seedling response to *Bgt* infection continued to produce significant deviations. In order to reduce the number of false positives (type 1 error), the threshold value for declaring the significance of SR QTL was raised to the more stringent 5.6 LOD value (corresponding to 0.000005 *p*) determined by the Bonferroni method [47].

**Figure 2:**
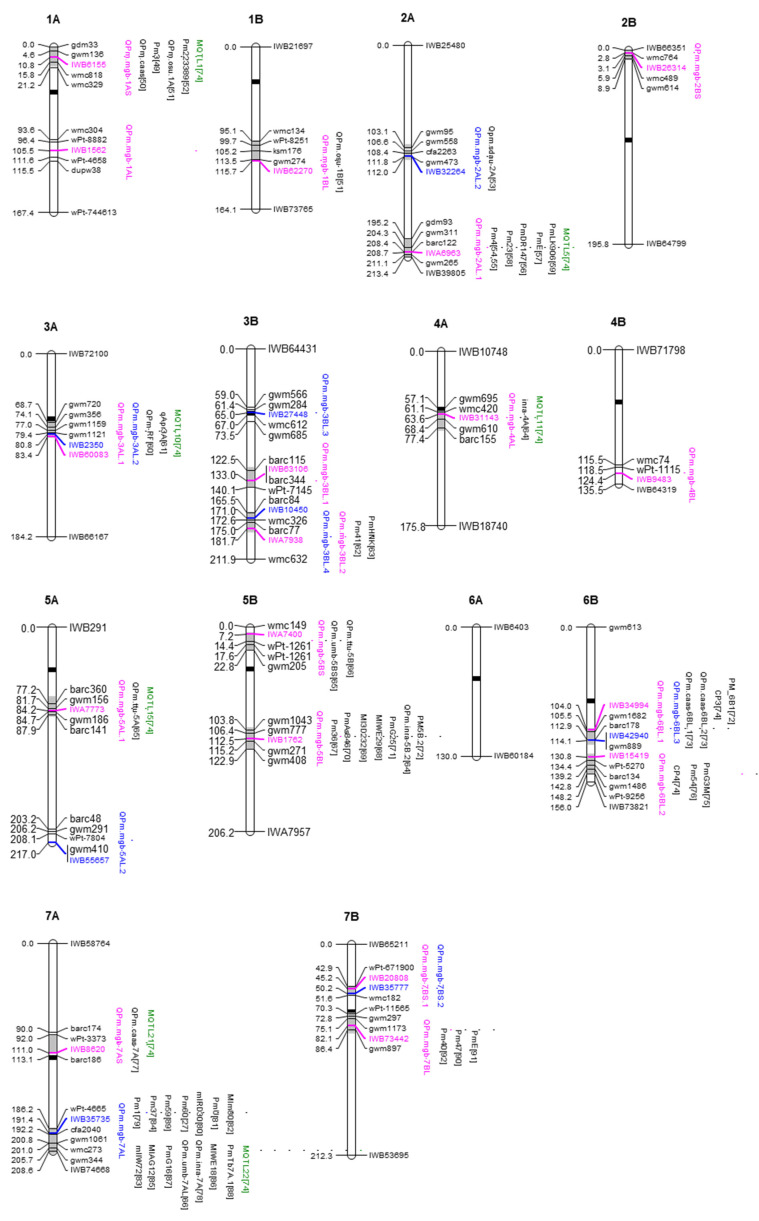
Chromosomal location on the durum wheat consensus map [42] of significant QTL for powdery mildew resistance detected in the current study in relation to previously mapped QTL. QTL-tagging SNPs identified by GWAS in the tetraploid wheat collection at seedlings and adult plants are in blue and red font, respectively. Previously mapped QTL on the confidence intervals (grey segment on the chromosome bar) are reported on the right side of each chromosome. The number in square brackets after each QTL indicates the relevant reference [49,50,51,52,53,54,55,56,57,58,59,60,61,62,63,64,65,66,67,68,69,70,71,72,73,74,75,76,77,78,79,80,81,82,83,84,85,86,87,88,89,90,91,92]. Meta-QTL reported by Marone et al. [74] are in green font. For better readability and comparison with simple sequence repeats (SSR)-based maps, some representative SSR markers are reported on the right side and the distance on the left side of each chromosome (1A-7B). Black segment on each chromosome bar represents the centromere.

The GWAS analysis detected 18 significant QTL for APR and 8 QTL for SR distributed on all chromosomes excluding 6A (Table 2 and Figure 2). Table 2 shows the map position of detected QTL according to the durum consensus map [42], the physical position of the tag-SNP marker for each QTL according to the durum wheat genome assembly [48], the frequency of the resistance and susceptibility alleles to *Bgt*, and the LOD score and the determination coefficient (R^2^) of each QTL. Four QTL for APR were detected on chromosomes 3AL, 3BL, 6BL and 7BS (*QPm.mgb-3AL.1, QPm.mgb-3BL.2, QPm.mgb-6BL.1, QPm.mgb-7BS.1*), co-located in the same confidence intervals of four QTL for SR (*QPm.mgb-3AL.2, QPm.mgb-3BL.4, QPm.mgb-6BL.3, QPm.mgb-7BS.2*), thus indicating different tightly linked loci or that the same loci might control APR and SR.

Thirteen alleles for APR and all 8 alleles for SR showed a lower frequency (from 5.1% for *QPm.mgb-5AL.2* to 27.3% for *QPm.mgb-2AL.1*) than the respective alleles for PM susceptibility (from 72.7 to 94.9%). Three alleles for APR (*QPm.mgb-4BL, QPm.mgb-5AL.1, QPm.mgb-5BS*) showed a higher frequency (from 85.5 to 95.5%) than their respective susceptibility alleles (from 5 to 15%). Two QTL for APR had alleles for resistance and susceptibility with the same frequency (*QPm.mgb-2AL.1 and QPm.mgb-7BL*). The phenotypic variation (R^2^) ranged from a minimum of 5.0% for *QPm.mgb-2AL.1* to 20.2% for *QPm.mgb-3BL.4*.

**Figure 3:**
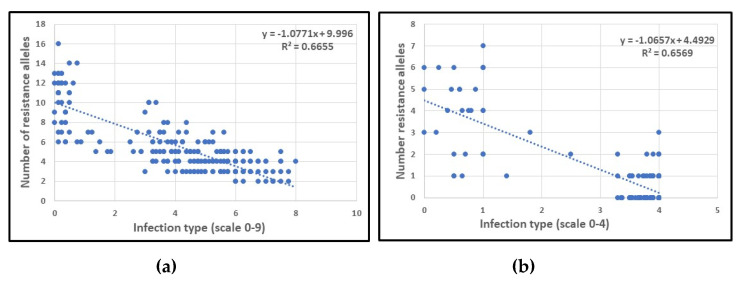
Linear regression of powdery mildew response of adult plants **(a)** and seedlings **(b)** to number of resistance alleles of QTL-tagging SNPs in each accession of the tetraploid wheat collection.

The number of resistance alleles of the detected QTL-tagging SNPs was significantly correlated with both APR and SR (correlation coefficients of −0.816 and 0.810, respectively). In general, wheat accessions with more favorable alleles showed stronger PM resistance. The scatter plots (Figure 3) clearly indicated a linear relationship between the number of favorable alleles and *Bgt* resistance.

### 2.3. Candidate Genes

The most prevalent types of fungal disease resistance genes in plants, including powdery mildew and rusts, contain nucleotide binding sites (NBS) and leucine-rich repeats (LRR) [93,94,95]. As candidate genes we considered the NBS-LRR genes present in the confidence intervals of the detected QTL for APR and SR. Maccaferri et al. [48] analyzed a large global tetraploid wheat collection of about 1856 accessions including different subspecies of *T. turgidum,* and the LD decay determined for each subspecies was 195 Kb for wild emmer, 1.4 Mb for domesticated emmer, 1.6 Mb for durum landraces and 4.5 Mb for cultivated durum wheat. In order to increase the likelihood of identifying potential candidate genes for PM resistance, the stringent LD decay value estimated for domesticated emmer (1.4 Mb, equivalent to a genetic distance of 0.35 cM) was considered as the confidence interval for each detected QTL. Table 3 reports the NBS-LRR candidate genes for the QTL for APR and SR, where marker position refers to the durum consensus map [42] and physical position to the genome assembly of durum wheat cv. Svevo.v1 [48]. Most of the putative candidate genes were classified as disease resistance protein RPM1, leucine-rich repeat receptor-like protein kinase, NBS-LRR disease resistance protein, F-box protein family-like. Interestingly, the marker IWB6155, associated with the APR QTL *QPm.mgb-1AS* at 10.8 cM on chromosome arm 1AS, was located within the gene *TRITD1Av1G004560* coding for a disease resistance protein RPM1. Two more SNP markers, IWB42940 and IWB35735, associated to the QTL *QPm.mgb-6BL.3* and *QPm.mgb-7AL* for SR, were found very close (3 and 10 Kb) to the genes *TRITD6Bv1G207200* and *TRITD7Av1G271480* coding for FBD-associated F-box protein and *Pm3*-like disease resistance protein, respectively.

## 3. Discussion

### 3.1. Detection of QTL for Bgt Resistance

The identification of new sources of PM resistance was carried out in two different greenhouse experiments by inoculating a tetraploid wheat collection with a natural mixture of *Bgt* races for the assessment of adult plant resistance and with the virulent *Bgt* isolate O2 for the seedling resistance. The bimodal-like distribution of seedling response to *Bgt* infection (Figure 1) suggested the presence of major genes controlling SR, while the distribution of APR indicated both the expression of major resistance genes and the segregation of quantitative loci with additive effects. The co-presence of qualitative and quantitative resistance in modern wheat cultivars was already detected in previous investigations on the effectiveness and environmental stability of quantitative PM resistance [96,97]. Major genes for SR usually confer PM resistance in all plant stages [98]. However, this type of resistance is overcome by the evolution of natural *Bgt* populations and the emergence of new virulent races, leading to the breakdown of PM resistance genes [99,100]. The decline in effectiveness of some *Pm* resistance genes was already observed in the eastern United States [7,8,9] and in China [10]. Quantitative resistance loci provide more durable and environmentally stable resistance than race-specific resistance genes, and therefore cultivars with quantitative resistance are normally selected in practical breeding [97,101,102,103].

In the current study, 18 QTL for APR were detected at the threshold of 0.001P (LOD ≥ 3.0) by the GWAS model MLM+Q+K selected as the most fitting model based on the deviations of observed -log_10_(*p*) values from the expected -log_10_(*p*) distributions (Q–Q plots, Figure S1). This threshold value is frequently used for QTL detection for several quantitative traits as well as for disease resistance [72,104,105]. Most QTL for APR (13 out of 18) were previously detected (Figure 2, Table S1), and this indicated the suitability of the GWAS model and of the threshold value used for claiming the QTL significance. However, a significant deviation (Q–Q plots, Figure S1) was observed for the SR. We used the same MLM+Q+K model for the SR too, but we applied a more stringent threshold value (LOD ≥ 5.6), determined by the Bonferroni method, in order to reduce the number of false-positive QTL. Using this threshold value, eight QTL for SR were detected, four of which mapped in the same confidence intervals where QTL for APR were mapped. Six of these QTL were mapped in previous studies (Figure 2, Table S3).

GWAS may result in false-positive (type 1 error) and false-negative (type 2 error) QTL as a function of population structure, relative kinship and threshold values used for claiming the significance of a marker-trait association [106,107]. In this study, 31 QTL for SR were identified at LOD ≥ 3.0, but 23 did not pass the stringent Bonferroni threshold value. These loci may have a greater proportion of false positives and were therefore excluded from the results reported in Table 2. However, increasing stringency could lead to the potential loss of sensitivity and to a higher number of false negatives [107,108]. Thus, for example, the marker IWB15419 was found to be associated with a QTL for SR at LOD = 4.7, but it was not reported in Table 2 as it did not pass the stringent experiment-wise Bonferroni threshold (LOD ≥ 5.6). This marker was found to be linked to the QTL *QPm.mgb-6BL.2* for APR (LOD 3.6) and was also previously detected [74,75,76]. Again, the marker IWB1762, significantly associated with the QTL *QPm.mgb-5BL* for APR on chromosome arm 5BL, was not considered to be associated to a QTL for SR as below the Bonferroni threshold value. We previously detected this QTL (*Pm36*) in a biparental segregating population derived from two backcross inbred lines [67]; this QTL was also detected by [64,68,69,70,71,72]. These examples might be cases of false negatives and this suggests that some of the lower-confidence QTL likely represent real associations, as was also reported by [105] in the detection of QTL for stem rust resistance. The lower-confidence QTL for PM resistance found in this study are currently under investigations to discriminate real associations from false-negative ones.

The 18 QTL for APR and 8 QTL for SR were distributed on 13 of the 14 *T. turgidum* chromosomes (Table 2 and Figure 2). Four QTL for APR located on chromosome arms 3AL, 3BL, 6BL and 7BS were identical or at least closely linked to four QTL for SR (Figure 2). The explained genetic variance (R^2^) of APR QTL was relatively small (range from 5.0% to 9.2%); similar values were reported in GWAS for stem rust [104,105,109] and powdery mildew [72,110,111]. The relatively high number of detected QTL for APR suggested that different mechanisms of PM resistance [17] could be involved in the diverse genetic background of the examined tetraploid wheat accessions.

The resistance alleles of three QTL for APR (*QPm.mgb-4BL, QPm.mgb-5AL.1* and *QPm.mgb-5BS*) showed a high frequency in the cultivated germplasm denoting the continuous work of durum breeders to improve this trait [6]. Eight out of nine *dicoccoides* accessions and 9 out of 19 *dicoccum* accessions were resistant at both adult and seedling stages, thus confirming several investigations reporting the utility of the wild and semi-domesticated gene pool for searching new source of resistance genes controlling wheat diseases [112,113,114]. In fact, 17 PM resistance genes have been already reported on chromosomes 2A, 2B, 3B, 4A, 5B, 6B and 7A of ssp. *dicoccoides* [17].

### 3.2. Comparison of Significant QTL with Previously Mapped QTL/Pm Resistance Genes

Several studies on QTL mapping for PM resistance in wheat have been published during the past few decades (see the recent review by Kang et al. [17]). The relationship between each QTL identified in the current study with previously mapped *Bgt* resistance QTL/genes is illustrated in detail in Figure 2 and Table S3. The wide chromosomal intervals consider that some previously reported PM resistance genes were localized in lower saturated SSR-based maps and the inherent limitations of the durum consensus map [42]. Thirteen out of 18 QTL for APR and six out of eight QTL for SR were mapped in similar positions where PM resistance QTL/genes were previously mapped (Figure 2 and Table S3), thus validating these QTL in different genetic backgrounds. These QTL likely represent alleles of previously mapped genes. An allelism test is required to determine which of the detected QTL are alleles of previous mapped genes or, alternatively, if they are novel resistance genes. Anyhow, these QTL can be considered stable QTL and useful for marker-assisted breeding programs.

The detected QTL were compared with the major *Pm* resistance genes that have been mapped or localized so far on wheat chromosomes. McIntosh et al. [15,16] catalogued reported 67 *Pm* resistance genes characterized for differential reactions to single *Bgt* races. The comparison showed that 12 QTL co-localize with *Pm* genes on chromosome arms 1AS (*QPm.mgb-1AS – Pm3*), 2AL (*QPm.mgb-2AL.1 – Pm4 – Pm23*), 3BS (*QPm.mgb-3BL.2 – QPm.mgb-3BL.4 Pm41*), 5BL (*QPm.mgb-5BL – Pm36*), 6BL (*QPm.mgb-6BL.2 – QPm.mgb-6BL.3 – Pm54*), 7AL (*QPm.mgb-7AL – Pm1 – Pm37 – Pm59 – Pm60*), 7BL (*QPm.mgb-7BL – Pm40 – Pm47*) (Figure 2). A further comparison was made with the meta-QTL for PM resistance reported by Marone et al. [74]. This study examined 23 publications describing a total of 101 QTL for powdery mildew resistance detected in 19 segregating populations of durum and bread wheat; 24 meta-QTL, comprising 2–6 initial QTL, distributed on 15 chromosomes of an SSR-based consensus map, were identified. Eight QTL detected in the current work were found in the confidence intervals of seven meta-QTL on chromosome arms 1AS (*QPm.mgb-1AS – MQTL1*), 2AL (*QPm.mgb-2AL.1 – MQTL5*), 3AL (*QPm.mgb-3AL.1 – QPm.mgb-3AL.1 – MQTL10*), 4AL (*QPm.mgb-4AL – MQTL11*), 4AL (*QPm.mgb-4AL – MQTL15*), 7AS (*QPm.mgb-7AS – MQTL21*), 7AL (*QPm.mgb-7AL – MQTL22*) (Figure 2).

While most of the QTL for *Bgt* resistance identified in the current study had been described previously (see Figure 2 for a detailed comparison), 5 out of 18 QTL for APR *(QPm.mgb-1AS, QPm.mgb-2BS, QPm.mgb-3BL.1, QPm.mgb-4BL, QPm.mgb-7BS.1)* detected on chromosome arms 1AS, 2BS, 3BL, 4BL and 7BS, and 3 QTL for SR (*QPm.mgb-3BL.3, QPm.mgb-5AL.2, QPm.mgb-7BS.2*) on 3BS, 5AL and 7BS were mapped on chromosome regions where, to our knowledge, no *Bgt* resistance genes were reported before. They might be novel *Bgt* resistance loci.

### 3.3. Candidate Genes for PM Resistance

In wheat, the linkage disequilibrium is high [48,115], and GWAS should be considered a first step to identify candidate genes [116]. Several hundreds of genes could be found in the confidence intervals of the significant markers linked to the trait of interest, and this makes impossible the identification of the causal genes [116]. We tried to find potential candidate genes for disease resistance in the gene sequences where the significant QTL-tagging SNPs were located, but we could not find candidate genes with obvious relations to disease resistance mechanisms. The only exception was the SNP marker IWB6155 significantly associated to *QPm.mgb-1AS* (Table 2), mapped at 10.8 cM on 1AS and physically located in the gene sequence *TRITD1Av1G004560* (9,960,116–9,964,051 bp) (Svevo reference genome assemby) [48]. This gene has an NBS-LRR domain and encodes for a disease resistance protein RPM1 [117]. In wheat and many other crops, disease resistance genes often encode for NBS-LRR receptors [93,94]. In fact, all the cloned PM resistance genes in wheat encode NBS-LRR proteins [18,19,20,21,22,23,24,25,26,27,28,29,30]. *QPm.mgb-1AS* was mapped to the genomic region where reside the well-known PM resistance gene *Pm3* [49] and the QTL *QPm.caas-1AS* [50], *QPm.osu-1A* [51] and *Pm223899* [52]. The coincidence of *QPm.mgb-1AS* with *Pm3* was excluded as the QTL-tagging SNP IWB71713 (intron variant), located inside the *Pm3* sequence, is physically located at 5,209,229–5,213,669 bp of the Svevo reference genome [48], and resides at 4.6 cM on the durum consensus map [42], while our candidate gene *TRITD1Av1G004560* is physically located at 9,960,116–9,964,051 bp and mapped at 10.8 cM in the durum consensus map [42]. The molecular characterization of the loci *QPm.caas-1AS* [50] and *QPm.osu-1A* [51] discovered that these QTL had a coincident location with *Pm3a*, while a linkage analysis placed *Pm223899* distally by the *Pm3* locus (0.3 cM) to an interval of about 831 Kb [52].

An additional QTL-tagging SNP IWB42940 associated to *QPm.mgb-6BL.3* was found closely linked (30,676 bp) to the NB-LRR resistance gene *TRITD6Bv1G207200* encoding for an FBD-associated F-box protein. In the confidence interval of *QPm.mgb-6BL.3,* the QTL *QPm.caas-6BL.2* [73], *CP3* [74] and *PM_6B1* [72] were previously mapped; the different associated markers do not allow to determine the coincidence of the loci or if they are different linked resistance loci. Another 11 candidate genes (Table 3), all encoding disease-related proteins, reside at a genetic distance lower than 0.35 cM and less than 1.400 kb.

### 3.4. Concluding Remarks

In the past, in different parts of the world, new *Bgt* races able to overcome the genetic resistance of cultivated germplasm and generating serious PM epidemics have been identified [7,8,9,10]. The validation of previously identified resistance genes and the novel QTL/genes for PM resistance identified in the current study can contribute to enriching the PM resistance source available to wheat breeders. The tetraploid wheat accessions can be easily crossed with cultivated common and durum wheat, thus contributing to diversifying the source of *Bgt* resistance genes. The SNP markers closely linked to PM resistance QTL/genes can be employed directly or transformed into KASP markers in order to accelerate marker-assisted breeding programs.

## 4. Materials and Methods

### 4.1. Plant Materials

A collection of 221 accessions of wild, semi-domesticated and cultivated tetraploid wheat (*Triticum turgidum* L., 2n = 4x = 28; AABB genome) from different countries of origin were used in this study to evaluate their resistance to powdery mildew. The panel included seven subspecies of *T. turgidum:* ssp. *durum* (125 old and modern cultivars of durum wheat), ssp. *turanicum* (20 accessions), ssp. *polonicum* (19 accessions), ssp. *turgidum* (18 accessions), ssp. *carthlicum* (12 accessions), ssp. *dicoccum* (18 accessions) and ssp. *dicoccoides* (9 accessions). The name/number of the genotypes, pedigree, country, and year of release are reported in Table S1.

### 4.2. Disease Evaluation

The tetraploid wheat collection was evaluated for adult-plant resistance (APR) and seedling resistance (SR) to powdery mildew in two distinct greenhouse experiments in 2015. A randomized complete block design with two replicates was used for the evaluation of APR, with plots consisting of 1 m rows, 30 cm apart, with 50 germinating seeds per plot. Ten plots of the disease reference cultivar Ciccio (PM susceptible) were placed randomly in each replicate. The experiment was carried out under controlled greenhouse conditions (temperature 15–25 °C and humidity 60–80%). A mixture of naturally occurred *Bgt* races was used as inoculum. The disease severity of each line was recorded when most of the accessions were at the anthesis stage and the infection on the susceptible check cv. Ciccio was at its maximum. The evaluation of plant response to *Bgt* was repeated after 15 days to limit the number of escapes, and the mean of the two records was subsequently considered for each replicate. The modified version of Cobb’s scale (0–9) as described by Peterson et al. [118] was used for scoring the disease severity. The scale 0–9 is based on PM severity and is expressed as the percentage of leaf surface area covered by mycelia where 0 = immune (no visible sign of infection); 1= 10% of leaf area covered by mycelia; 2 = 20%; 3 = 30%; 4= 40%; 5 = 50%; 6 = 60%; 7 = 70%; 8 = 80%; 9 = dal 90% al 100. The average of the two replicates of each accession are reported on Table S1.

The PM evaluation at seedling stage was carried out under controlled greenhouse conditions with the *Bgt* isolate O2 [119]. Twelve seeds of each accession were sown in round pots with a diameter of 15 cm and grown to the two-leaf stage. Inoculation was performed by blowing the spores into a plastic tower at a density of 4×10^3^ conidia cm^−2^. Twelve days post-inoculation, when the susceptible cv. Ciccio showed fully developed disease symptoms, 6–8 seedlings of each genotype were individually assessed for the infection type (IT) according to the 0–4 Stakman scale [44] not encompassing symbols (e.g.; +), where ITs of 0 = immune (no visible sign of infection); 1 = no mycelia and presence of resistance spots; 2 = 10–20% of leaf area covered by mycelia; 3= 20–50% of leaf area covered by mycelia; 4 = 50–100% of leaf area covered by mycelia). An average of 6–8 seedling values of each line were reported on Table S1.

### 4.3. DNA Extraction and SNP Marker Analysis.

Genomic DNA from the 221 lines was isolated from freeze-dried leaf tissue following the protocol by Dellaporta [120] and checked on 1.5% denaturing agarose gel. A total of 50 ng of genomic DNA of each accession was sent to TraitGenetics GmbH (Gatersleben, Germany) [121] for genotyping by the wheat 90K Infinium iSelect array developed by Illumina CSProR (San Diego, CA, USA) and described by Wang et al. [46]. The genotyping assays used an Illumina iScan reader and analyzed using Genome Studio software version 2011.1 (Illumina, Inc., San Diego, CA, USA).

### 4.4. Phenotypic Data Analysis and QTL Detection

Statistical analyses of APR and SR data were carried out using the software MSTAT-C. The Shapiro–Wilks test [45] was used to test the normality of the APR and SR distributions. Genetic diversity and population structure of the tetraploid wheat collection have been previously analyzed by SSR [115] and SNP markers [122], to which references should be made for a detailed description. SNP markers with a minimum allele frequency (MAF) of less than 5% and with >20% missing data points were not included in the data matrix. The durum wheat consensus map [42] was used as a reference map for chromosome localization and map positions of SNP markers associated to QTL. Unmapped markers were also removed and a total of 19,393 mapped markers were used for the subsequent genome-wide association study (GWAS) analysis. Mean values of 6–8 seedlings inoculated with the *Bgt* isolate O2 and the mean values across replicates of APR were used in the GWAS using the software TASSEL v.5 [123]. Original seedling resistance values, expressed according to the Stackman disease scale 0–4 [44], were converted to a 0–9 linear disease scale, where 0 = immune and 9 = very susceptible, as described by Zhang et al. [124] to meet the data format required for association analysis. The IT were converted as follows: 0 = 0; 0.01 − 0.70 = 1; 0.71 − 1.00 = 2; 1.01 − 1.30 = 3; 1.31 − 1.70 = 4; 1.71 − 2.00 = 5; 2.01 − 2.30 = 6; 2.31 − 2.70 = 7; 2.71 − 3.00 = 8; 3.01 − 4.00 = 9. The converted datasets were then used for the GWAS. Most lines from the tetraploid wheat collection were skewed towards the resistance values to PM and the frequency distribution deviated from the normal curve. The original data were transformed by arcsin, radq and log, but the transformed results do not substantially differ from the presented analysis (results not shown). The association between SNP markers and PM resistance was tested by four models implemented in TASSEL, that is a) general linear model (GLM), b) GLM including the Q-matrix derived from the principal component analysis (GLM+Q), c) mixed linear model based on the kinship-matrix (MLM+K), and d) MLM including both the K-matrix and the Q-matrix (MLM+K+Q). The quantile–quantile (Q–Q) plots showing the observed *p*-values and the expected *p*-values were used for model comparison and selection of the optimal model for the association mapping analysis. The model MLM + K+Q with 1000 permutations fit well for APR (Figure S1), and the threshold of *p* value at 0.001 (−log10(*p*) ≥ 3) was set up to declare significant marker-trait associations. However, the observed *p*-values for SR deviated from the expected *p*-values by the four examined models. A more stringent threshold value determined by the Bonferroni method (= 0.05/m where m is the total number of markers) (*p* = 0.00005 equivalent to LOD = 5.6) was used to reduce the chances of obtaining false-positive results (type I errors) for SR. QTL were designated according to the catalogue of gene symbols for wheat [15].

Comparison of QTL locations detected in the current study with previously reported QTL/*Bgt* genes was carried out considering confidence intervals of 10–20 cM to take into account that some of the QTL reported in the literature were identified in biparental populations characterized mainly with SSR markers and with low resolution maps. Graphical representation of linkage groups and QTL was determined by the MapChart 2.2 software.

### 4.5. Candidate Genes

Putative candidate genes associated with APR and SR were identified using the genome assembly of *T. turgidum* cv. Svevo [48]. As many fungal disease resistance genes, including powdery mildew and rusts, are characterized by nucleotide binding sites (NBS) and leucine rich repeats (LRR) [13,93,94], all the NBS-LRR genes reported in the Svevo genome assembly were downloaded from the Ensamble Plants website [117]. Putative candidate genes were considered the NBS-LRR genes associated to the QTL-tagging SNPs within a physical distance of 1400 Kb (equivalent to a genetic distance of 0.35 cM) as determined by the LD decay in a collection of ssp. *dicoccum* accessions [95].

## Figures and Tables

**Table 1:** Summary of phenotypic data of 221 tetraploid wheat accessions evaluated for their reaction to *Blumeria graminis* f. sp. *tritici* at adult plant and seedling stages. All lines were classified into two groups with resistant lines (R) scoring 0–2 at seedlings and adult plants, and susceptible lines (S) scoring 2.1–4.0 for seedlings and 2.1–9.0 for adult plants. SD = standard deviation. RR = Resistant accessions at seedling and adult plant stages.

*Triticum turgidum* Subspecies	N. of Accessions	Adult Plants (Infection Type Scale 0–9)						Seedlings (Infection Type Scale 0–4)						RR
		Mean	SD	Min.	Max.	R	S	Mean	SD	Min.	Max.	R	S	
*durum*	125	4.9	1.7	0.0	7.8	10	115	3.8	0.7	0.5	4.0	5	120	3
*turanicum*	20	5.6	1.1	3.1	8.0	0	20	3.9	0.2	3.4	4.0	0	20	0
*polonicum*	19	2.2	2.2	0.0	7.5	10	9	3.4	1.2	0.5	4.0	3	16	3
*turgidum*	18	3.7	0.4	0.1	5.6	3	15	3.9	0.2	3.5	4.0	0	18	0
*carthlicum*	12	3.1	2.3	0.1	6.3	4	8	2.9	1.5	0.5	4.0	4	8	3
*dicoccum*	18	0.8	1.6	0.0	5.6	15	3	2.0	1.7	0.0	4.0	10	8	9
*dicoccoides*	9	0.6	1.0	0.0	3.1	8	1	1.2	1.2	0.0	4.0	8	1	8
*Whole collection*	221	4.0	2.3	0.0	8.0	50	171	3.5	1.1	0.0	4.0	30	191	26

**Table 2:** Quantitative trait loci (QTL) for adult plant resistance and seedling (isolate O2) resistance to *Blumeria graminis* f. sp. *tritici* in a tetraploid wheat collection. Marker position is referred to the durum wheat consensus map [42] and physical position to the genome assembly of *Triticum turgidum* ssp. *durum* cv. Svevo.v1 [48].

QTL	Marker	Chrom.	Genetic Position (cM)	Physical Position (bp)	SNP Allele		Allele Frequency		*p* Value	R^2^ (%)
					R	S	R	S	-log10(*p*)	
**Adult plants**										
*QPm.mgb-1AS*	IWB6155	1AS	10.8	9,960,116	A	C	31	157	3.0	5.6
*QPm.mgb-1AL*	IWB1562	1AL	105.5	535,547,520	A	G	35	184	3.4	5.9
*QPm.mgb-1BL*	IWB62270	1BL	115.7	560,384,200	A	G	60	160	3.4	6.0
*QPm.mgb-2AL.1*	IWA6963	2AL	208.7	774,519,768	A	G	100	116	3.0	5.0
*QPm.mgb-2BS*	IWB26314	2BS	3.1	4,844,683	C	T	15	202	3.4	5.9
*QPm.mgb-3AL.1*	IWB60083	3AL	83.4	549,634,714	G	A	38	183	4.0	7.2
*QPm.mgb-3BL.1*	IWB63106	3BL	133.0	731,241,583	G	T	68	119	4.2	9.2
*QPm.mgb-3BL.2*	IWA7938	3BL	181.7	797,223,552	G	A	49	164	3.8	6.9
*QPm.mgb-4AL*	IWB31143	4AL	63.6	555,057,750	A	G	24	196	3.0	5.0
*QPm.mgb-4BL*	IWB9483	4BL	124.4	674,637,578	C	T	202	13	3.2	5.6
*QPm.mgb-5AL.1*	IWA7773	5AL	84.2	431,817,017	A	G	210	10	3.1	5.2
*QPm.mgb-5BS*	IWA7400	5BS	7.2	11,753,122	C	T	188	32	3.0	5.2
*QPm.mgb-5BL*	IWB1762	5BL	112.5	545,353,694	T	C	36	170	3.6	6.5
*QPm.mgb-6BL.1*	IWB34994	6BL	104.0	569,080,772	T	C	26	182	3.0	5.5
*QPm.mgb-6BL.2*	IWB15419	6BL	130.8	666,669,725	C	A	13	201	3.6	6.4
*QPm.mgb-7AS*	IWB8620	7AS	111.0	224,203,539	T	C	26	192	3.4	5.9
*QPm.mgb-7BS.1*	IWB20808	7BS	45.2	71,150,010	T	C	17	204	3.5	6.2
*QPm.mgb-7BL*	IWB73442	7BL	82.1	411,065,432	A	G	111	108	3.7	6.5
**Seedlings**										
*QPm.mgb-2AL.2*	IWB32264	2AL	112.0	510,733,662	A	G	12	207	5.9	11.6
*QPm.mgb-3AL.2*	IWB2350	3AL	80.8	540,811,043	G	A	15	205	6.4	12.5
*QPm.mgb-3BL.3*	IWB27448	3BS	65.0	149,866,560	A	G	23	196	6.2	12.0
*QPm.mgb-3BL.4*	IWB10450	3BL	171.0	787,574,962	C	T	24	192	9.5	20.2
*QPm.mgb-5AL.2*	IWB55657	5AL	217.0	666,966,392	G	A	11	207	7.0	13.8
*QPm.mgb-6BL.3*	IWB42940	6BL	114.1	642,292,418	T	C	15	183	6.2	14.2
*QPm.mgb-7AL*	IWB35735	7AL	191.4	705,221,250	C	T	50	168	6.4	12.6
*QPm.mgb-7BS.2*	IWB35777	7BS	50.2	91,214,298	T	C	30	184	6.8	14.4

R = Resistance allele; S = Susceptibility allele; R^2^(%) = Percentage of phenotypic variation explained by the single nucleotide polymorphism (SNP) marker.

**Table 3:** Candidate genes for the quantitative trait loci (QTL) for seedlings and adult plants resistance to *Blumeria graminis* f. sp. *tritici* in a tetraplod wheat collection. Physical position to the genome assembly of *Triticum turgidum* ssp. *durum* cv. Svevo.v1 [48].

QTL-Tagging SNP	Chr.	Gene-ID	Physical Position (bp)	Gene Annotation
**Adult plants**				
IWB6155	1AS	TRITD1Av1G004560	9,960,116–9,964,051	Disease resistance protein RPM1
IWB1562	1AL	TRITD1Av1G205580	535,763,118–535,766,921	Disease resistance protein (NBS-LRR class) family
IWA6963	2AL	TRITD2Av1G295560	774,749,507–774,761,574	NBS-LRR resistance-like protein
IWB26314	2BS	TRITD2Bv1G002930	5,515,403–5,637,942	LRR receptor-like protein kinase family protein
IWB63106	3BL	TRITD3Bv1G240250	731,784,051–731,787,435	Disease resistance family protein
IWA7938	3BL	TRITD3Bv1G266330	796,494,720–796,500,214	NBS-LRR-like resistance protein
IWA7773	5AL	TRITD5Av1G155690	433,018,841–433,022,231	Receptor protein kinase, putative
IWA7400	5BS	TRITD5Bv1G004470	10,953,479–1,0956,756	LRR receptor-like protein kinase
IWB20808	7BS	TRITD7Bv1G026230	72,100,728–72,102,713	Receptor-like kinase, putative
**Seedlings**				
IWB2350	3AL	TRITD3Av1G193390	540,583,802–540,591,916	Receptor-like protein kinase
IWB10450	3BL	TRITD3Bv1G261700	787,289,090–787,289,936	F-box protein family-like
IWB42940	6BL	TRITD6Bv1G207200	642,323,094–642,579,232	FBD-associated F-box protein
IWB35735	7AL	TRITD7Av1G271480	705,323,881–705,327,860	Pm3-like disease resistance protein

## Supplementary Materials

Supplementary materials can be found at https://www.mdpi.com/1422-0067/21/21/7910/s1. Table S1: List of accessions of *Triticum turgidum* subspecies included in the tetraploid wheat collection and infection type values for resistance to powdery mildew at seedlings and adult plants. Table S2. Number of SNP markers polymorphic in the tetraploid wheat collection and map position according to the durum wheat consensus map [42] used as reference map. Table S3. Comparison of significant QTL for powdery mildew resistance at seedlings and adult plants to previously reported *Pm* genes/ QTL based on map positions of the consensus durum map [42]. Figure S1. Genome-wide association analysis for adult plant resistance and seedling resistance in a tetraploid wheat collection. Quantile-quantile (Q-Q) plots of the observed –log10 (*p*) values (y axes) against the expected distribution of –log10 (*p*) values (x axes) for the models: (a) GLM, (b) GLM+Q, (c) MLM+K, (d) MLM+K+Q. Red line = Adult plants. Blue line = seedlings

## Abbreviations

QTL	Quantitative Trait Loci
APR	Adult Plant Resistance
SR	Seedling resistance
Bgt	Blumeria graminis f. sp. tritici
IT	Infection Type
PM	Powdery Mildew
GLM	General Linear Model
MLM	Mixed Linear Model
Q	Q matrix defined by Principal Components
K	Kinship matrix
Q–Q	Quantile-Quantile plot
LD	Linkage Disequilibrium
SNP	Single Nucleotide Polymorphism
MAF	Minor Allele Frequency
NBS-LRR	Nucleotide Binding Sites and Leucine-Rich Repeat proteins

## References

[1] FAO. http://www.fao.org/faostat/en/#data.

[2] Tilman D., Balzer C., Hill J., Befort B.L. (2011). Global food demand and the sustainable intensification of agriculture. Proc. Natl. Acad. Sci. USA.

[3] Bailey-Serres J., Parker J.E., Ainsworth E.A., Oldroyd G., Schroeder J.I. (2019). Genetic strategies for improving crop yields. Nature.

[4] Mehta Y.R. (2014). Wheat and Wheat Production Constraints. Wheat Diseases and Their Management.

[5] Bennett F.G.A. (1984). Resistance to powdery mildew in wheat: A review of its use in agriculture and breeding programmes. Plant Pathol..

[6] Summer R.W., Brown J.K.M. (2013). Constraints on breeding for disease resistance in commercially competitive wheat cultivars. Plant Pathol..

[7] Niewoehner A.S., Leath S.N. (1998). Virulence of *Blumeria graminis* f. sp. *tritici* on winter wheat in the Eastern United States. Plant Dis..

[8] Parks R., Carbone I., Murphy J.P., Marshall D., Cowger C. (2008). Virulence structure of the Eastern U.S. wheat powdery mildew population. Plant Dis..

[9] Cowger C., Parka R., Marshall O. (2009). Appearance of powdery mildew of wheat caused by *Blumeria graminis* f. sp*. tritici* on *Pm17*-bearing cultivars in North Carolina. Plant Dis..

[10] Wang Z.L., Li L.H., He Z.H., Duan X.Y., Zhou Y.L., Chen X.M., Lillemo M., Singh R.P., Wang H., Xia X.C. (2005). Seedling and adult plant resistance to powdery mildew in chinese bread wheat cultivars and lines. Plant Dis..

[11] Wallwork H., Walters D. (2009). The use of host plant resistance in disease control. Disease Control in Crops: Biological and Environmentally Friendly Approaches.

[12] Mundt C.C. (2014). Durable resistance: A key to sustainable management of pathogens and pests. Infect. Genet. Evol..

[13] Dangl J.L., Jones J.D.G. (2001). Plant pathogens and integrated defence responses to infection. Nature.

[14] Flor H.H. (1971). Current status of the gene-for-gene concept. Annu. Rev. Phytopathol..

[15] McIntosh R., Yamazaki Y., Dubcovsky J., Rogers W.J., Morris C., Appels R., Xia X.C. Catalogue of gene symbols for wheat. http://shigen.nig.ac.jp/wheat/komugi/genes/symbolClassList.jsp.

[16] McIntosh R.A., Dubcovsky J., Rogers W.J., Xia X.C., Raupp W.J. (2019). Catalogue of gene symbols for wheat. Suppl. Ann. Wheat Newsl..

[17] Kang Y., Zou M., Merry A., Barry K. (2020). Mechanisms of powdery mildew resistance of wheat–a review of molecular breeding. Plant Pathol..

[18] Yahiaoui N., Srichumpa P., Dudler R., Keller B. (2004). Genome analysis at different ploidy levels allows cloning of the powdery mildew resistance gene *Pm3b* from hexaploid wheat. Plant J..

[19] Krattinger S.G., Lagudah E.S., Spielmeyer W., Singh R.P., Huerta-Espino J., McFadden H., Bossolini E., Selter L.L., Keller B. (2009). A putative ABC transporter confers durable resistance to multiple fungal pathogens in wheat. Science.

[20] Hurni S., Brunner S., Buchmann G., Herren G., Jordan T., Krukowski P., Wicker T., Yahiaoui N., Mago R., Keller B. (2013). Rye *Pm8* and wheat *Pm3* are orthologous genes and show evolutionary conservation of resistance function against powdery mildew. Plant J..

[21] Moore J.W., Herrera-Foessel S., Lan C., Schnippenkoetter W., Ayliffe M., Huerta-Espino J., Lillemo M., Viccars L., Milne R., Periyannan S. (2015). Recently evolved hexose transporter variant confers resistance to multiple pathogens in wheat. Nat. Gen..

[22] Sánchez-Martín J., Steuernagel B., Ghosh S., Herren G., Hurni S., Adamski N. (2016). Rapid gene isolation in barley and wheat by mutant chromosome sequencing. Gen. Biol..

[23] Cao A., Xing L., Wang X., Yang X., Wang W., Sun Y., Qian C., Ni J., Chen Y., Liu D. (2011). Serine/threonine kinase gene stpk-v, a key member of powdery mildew resistance gene *Pm21*, confers powdery mildew resistance in wheat. Proc. Natl. Acad. Sci. USA.

[24] He H., Zhu S., Zhao R., Jiang Z., Ji Y., Ji J., Qiu D., Li H., Bie T. (2018). *Pm21,* encoding a typical CC-NBS-LRR protein, confers broad-spectrum resistance to wheat powdery mildew disease. Mol. Plant.

[25] Xing L., Hu P., Liu J., Witek K., Zhou S. (2018). *Pm21* from *Haynaldia Villosa* encodes a CC-NBS-LRR protein conferring powdery mildew resistance in wheat. Mol. Plant.

[26] Singh S.P., Hurni S., Ruinelli M., Brunner S., Sanchez-Martin J., Krukowski P., Peditto D., Buchmann G., Zbinden H., Keller B. (2018). Evolutionary divergence of the rye *Pm17* and *Pm8* resistance genes reveals ancient diversity. Plant Mol. Biol..

[27] Zou S., Wang H., Li Y., Kong Z., Tang D. (2018). The NB-LRR gene *Pm60* confers powdery mildew resistance in wheat. New Phytol..

[28] Xie J., Guo G., Wang Y., Hu T., Wang L. (2020). A Rare single nucleotide variant in *Pm5e* Confers powdery mildew resistance in common wheat. New Phytol..

[29] Lu P., Guo L., Wang Z., Li B., Li J., Li Y., Qiu D., Shi W., Yang L., Wang N. (2020). A rare gain of function mutation in a wheat tandem kinase confers resistance to powdery mildew. Nat. Commun..

[30] Li M., Dong L., Li B., Wang Z., Xie J., Qiu D., Li Y., Shi W., Yang L., Wu Q. (2020). A CNL protein in wild emmer wheat confers powdery mildew resistance. New Phytol..

[31] Jorgensen J.H. (1972). Discovery, characterization and exploitation of *Mlo* powdery mildew resistance in barley. Euphytica.

[32] Devoto A., Hartmann H.A., Piffanelli P., Elliott C., Simmons C., Taramino G. (2003). Molecular phylogeny and evolution of the plant-specific seven-transmembrane MLO family. J. Mol. Evol..

[33] Varallyay E., Giczey G., Burgyan J. (2012). Virus-induced gene silencing of *Mlo* genes induces powdery mildew resistance in *Triticum aestivum*. Arch. Virol..

[34] Lyngkjaer M.F., Newton A.C., Atzema J.L., Baker S.J. (2000). The barley *mlo*-gene: An important powdery mildew resistance source. Agronomie.

[35] Wang Y., Cheng X., Shan Q., Zhang Y., Liu J., Gao C., Qiu J.L. (2014). Simultaneous editing of three homoeoalleles in hexaploid bread wheat confers heritable resistance to powdery mildew. Nat. Biotechnol..

[36] Acevedo-Garcia J., Spencer D., Thieron H., Reinstädler A., Hammond-Kosack K., Phillips A.L., Panstruga R. (2017). *Mlo*-based powdery mildew resistance in hexaploid bread wheat generated by a non-transgenic TILLING approach. Plant Biotechnol. J..

[37] Lander E.S.B., Botstein D. (1989). Mapping mendelian factors underlying quantitative traits using RFLP linkage maps. Genetics.

[38] Tankley S.D. (1993). Mapping Polygenes. Ann. Rev. Gen..

[39] Rafalski J.A. (2010). Association genetics in crop improvement. Curr. Opin. Plant Biol..

[40] Astle W., Balding D.J. (2009). Population structure and cryptic relatedness in genetic association studies. Statist. Sci..

[41] Gupta P.K., Kulwal P.L., Jaiswal V. (2019). Association mapping in plants in the post-GWAS genomics era. Adv. Genet..

[42] Maccaferri M., Ricci A., Salvi S., Milner S.G., Noli E., Martelli P.L., Casadio R., Akhunov E., Scalabrin S., Vendramin V. (2015). A high-density, SNP-based consensus map of tetraploid wheat as a bridge to integrate durum and bread wheat genomics and breeding. Plant Biotechnol. J..

[43] Rong J.K., Millet E., Manisterski J., Feldman M. (2000). A new powdery mildew resistance gene: Introgression from wild emmer into common wheat and RFLP-based mapping. Euphytica.

[44] Stakman E.C., Stewart D.M., Loegering W.Q. (1962). Identification of physiologic races of *Puccinia graminis* var. *tritici*. USDA-ARS Bull E-617 Ed US Gov Print Office.

[45] Shapiro S.S., Wilk M.B. (1965). An analysis of variance test for normality (Complete Samples). Biometrika.

[46] Wang S., Wong D., Forrest K., Allen A., Chao S. (2014). Characterization of polyploid wheat genomic diversity using a high-density 90,000 SNP array. Plant Biotechnol. J..

[47] Sedgwick P. (2012). Multiple significance tests: The Bonferroni correction. BMJ.

[48] Maccaferri M., Harris N.S., Twardziok S.O., Pasam R.K., Gundlach H. (2019). Durum wheat genome highlights past domestication signatures and future improvement targets. Nat. Genet..

[49] Zeller F.J., Lutz J., Stephan U. (1993). Chromosome location of genes for resistance to powdery mildew in common wheat (*Triticum aestivum* L.) Mlk and other alleles at the *Pm3 l*ocus. Euphytica.

[50] Liang S.S., Suenaga K., He Z.H., Wang Z.L., Liu H.Y., Wang D.S., Singh R.P., Sourdille P., Xia X.C. (2006). Quantitative trait loci mapping for adult-plant resistance to powdery mildew in bread wheat. Phytopathology.

[51] Chen Y., Hunger R.M., Carver B.F., Zhang H., Yan L. (2009). Genetic characterization of powdery mildew resistance in u.s. hard winter wheat. Mol. Breed..

[52] Li G., Carver B.F., Cowger C., Bai G., Xu X. (2018). *Pm223899*, a new recessive powdery mildew resistance gene identified in Afghanistan landrace PI. Theor. Appl. Genet..

[53] Qu C., Guo Y., Kong F., Zhao Y., Li H., Li S. (2018). Molecular mapping of two quantitative trait loci for adult-plant resistance to powdery mildew in common wheat (*Triticum aestivum* L.). Crop Protect..

[54] Ullah K.N., Li N., Shen T. (2018). Fine mapping of powdery mildew resistance gene *Pm4e* in bread wheat (*Triticum aestivum* L.). Planta.

[55] Ma Z.Q., Sorrells M.E., Tanksley S.D. (1994). RFLP markers linked to powdery mildew resistance genes *Pm1, Pm2, Pm3,* and *Pm4* in wheat. Genome.

[56] Zhu Z., Zhou R., Kong X., Dong Y., Jia J. (2005). Microsatellite markers linked to 2 powdery mildew resistance genes introgressed from *Triticum carthlicum* accession PS5 into common wheat. Genome.

[57] Zhou R., Zhu Z., Kong X., Huo N., Tian Q., Li P., Jin C., Dong Y., Jia J. (2005). Development of wheat near-isogenic lines for powdery mildew resistance. Theor. Appl. Genet..

[58] Hao Y., Liu A., Wang Y., Feng D., Gao J., Li X., Liu S., Wang H. (2008). *Pm23*: A new allele of Pm4 located on chromosome 2AL in wheat. Theor. Appl. Genet..

[59] Niu J.S., Wang B.Q., Wang Y.H., Cao A.Z., Qi Z.J., Shen T.M. (2008). Chromosome location and microsatellite markers linked to a powdery mildew resistance gene in wheat line “Lankao 90(6)”. Plant Breed..

[60] Mingeot D., Chantret N., Baret P.V., Dekeyser A., Boukhatem N., Sourdille P., Doussinault G., Jacquemin J.M. (2002). Mapping QTL involved in adult plant resistance to powdery mildew in the winter wheat line RE714 in two susceptible genetic backgrounds. Plant Breed..

[61] Bougot Y., Lemoine J., Pavoine M.T., Guyomar’ch H., Gautier V., Muranty H., Barloy D. (2006). A major QTL effect controlling resistance to powdery mildew in winter wheat at the adult plant stage. Plant Breed..

[62] Zhang K.P., Zhao L., Hai Y., Chen G.F., Tian J.C. (2008). QTL Mapping for adult-plant resistance to powdery mildew, lodging resistance, and internode length below spike in wheat. Acta Agron. Sin..

[63] Li G., Fang T., Zhang H., Xie C., Li H., Yang T., Nevo E., Fahima T., Sun Q., Liu Z. (2009). Molecular identification of a new powdery mildew resistance gene *Pm41* on chromosome 3BL derived from wild emmer (*Triticum turgidum* var. *dicoccoides*. Theor. Appl. Genet..

[64] Xu H., Yao G., Xiong L., Yang L., Jiang Y., Fu B., Zhao W., Zhang Z., Zhang C., Ma Z. (2008). Identification and mapping of *Pm2026*: A recessive powdery mildew resistance gene in an einkorn (*Triticum monococcum* L.) accession. Theor. Appl. Genet..

[65] Jakobson I., Peusha H., Timofejeva L., Järve K. (2006). Adult plant and seedling resistance to powdery mildew in a *Triticum aestivum x Triticum militinae* hybrid line. Theor. Appl. Genet..

[66] Lillemo M., Bjørnstad A., Skinnes H. (2012). Molecular mapping of partial resistance to powdery mildew in winter wheat cultivar Folke. Euphytica.

[67] Blanco A., Gadaleta A., Cenci A., Carluccio A.V., Abdelbacki A.M.M., Simeone R. (2008). Molecular mapping of the novel powdery mildew resistance gene *Pm36* introgressed from *Triticum turgidum* var. *dicoccoides* in durum wheat. Theor. Appl. Genet..

[68] Zhang L.S., Hua W., Guan H.Y., Li G.Q., Xie C.J., Yang Z.M., Sun Q.X., Liu Z.Y. (2009). Molecular mapping of powdery mildew resistance gene *MlWE29* in wheat originated from wild emmer (*Triticum turgidum* var. *dicoccoides*). Acta Agron. Sin..

[69] Zhang H., Guan H., Li J., Zhu J., Xie C., Zhou Y., Duan X., Yang T., Sun Q., Liu Z. (2010). Genetic and comparative genomics mapping reveals that a powdery mildew resistance gene *Ml3D232* originating from wild emmer co-segregates with an NBS-LRR analog in common wheat (*Triticum aestivum* L). Theor. Appl. Genet..

[70] Xue F., Ji W., Wang C., Zhang H., Yang B. (2012). High-density mapping and marker development for the powdery mildew resistance gene *PmAS846* derived from wild emmer wheat (*Triticum turgidum* var. *dicoccoides*). Theor. Appl. Genet..

[71] Alam M.A., Mandal M.S.N., Wang C., Ji W. (2013). Chromosomal location and SSR markers of a powdery mildew resistance gene in common wheat line N. Afr. J. Micro. Res.

[72] Kang Y., Barry K., Cao F., Zhou M. (2020). Genome-wide association mapping for adult resistance to powdery mildew in common wheat. Mol. Biol. Rep..

[73] Asad M.A., Bai B., Lan C.X., Yan J., Xia X.C., Zhang Y., He Z.H. (2012). Molecular mapping of quantitative trait loci for adult-plant resistance to powdery mildew in Italian wheat cultivar Libellula. Crop Past. Sci..

[74] Marone D., Russo M.A., Laidò G., De Vita P., Papa R., Blanco A., Gadaleta A., Rubiales D., Mastrangelo A.M. (2013). Genetic basis of qualitative and quantitative resistance to powdery mildew in wheat: From consensus regions to candidate genes. BMC Genom..

[75] Xie W., Ben-David R., Zeng B., Distelfeld A., Roder M.S., Dinoor A., Fahima T. (2012). Identification and characterization of a novel powdery mildew resistance gene *PmG3M* derived from wild emmer wheat, *Trit Dicoccoid*. Theor. Appl. Genet..

[76] Hao Y., Parks R., Cowger C., Chen Z., Wang Y. (2015). Molecular characterization of a new powdery mildew resistance gene *Pm54* in soft red winter wheat. Theor. Appl. Genet..

[77] Lan C., Liang S., Wang Z., Yan J., Zhang Y., Xia X., He Z. (2009). Quantitative trait loci mapping for adult-plant resistance to powdery mildew in chinese wheat cultivar Bainong. Phytopathology.

[78] Chantret N., Sourdille P., Röder M., Tavaud M., Bernard M., Doussinault G. (2000). Location and mapping of the powdery mildew resistance gene MlRE and detection of a resistance QTL by bulked segregant analysis (BSA) with microsatellites in wheat. Theor. Appl. Genet..

[79] Singrun C.H., Hsam S.L.K., Hartl L., Zeller J., Mohler V. (2003). Powdery mildew resistance gene *Pm22* in cultivar Virest is a member of the complex *Pm1* locus in common wheat (*Triticum aestivum* L. em Thell.). Theor. Appl. Genet..

[80] Singrün C., Hsam S.L., Zeller F.J., Wenzel G., Mohler V. (2004). Localization of a novel recessive powdery mildew resistance gene from common wheat line RD30 in the terminal region of chromosome 7AL. Theor. Appl. Genet..

[81] Qiu Y.C., Zhou R.H., Kong X.Y., Zhang S.S., Jia J.Z. (2005). Microsatellite mapping of a *Triticum urartu* derived powdery mildew resistance gene transferred to common wheat *Triticum aestivum* L. Theor. Appl. Genet..

[82] Yao G., Zhang J., Yang L., Xu H., Jiang Y., Xiong L., Zhang C., Zhang Z., Ma Z., Sorrells M.E. (2007). Genetic mapping of two powdery mildew resistance genes in einkorn (*Triticum monococcum* L.) accessions. Theor. Appl. Genet..

[83] Ji X., Xie C., Ni Z., Yang T., Nevo E., Fahima T., Liu Z., Sun Q. (2008). Identification and genetic mapping of a powdery mildew resistance gene in wild emmer *Triticum dicoccoides* accession IW72 from Israel. Euphytica.

[84] Perugini L.D., Murphy J.P., Marshall D., Brown-Guedira G. (2008). *Pm37*, a new broadly effective powdery mildew resistance gene from *Triticum timopheevii*. Theor. Appl. Genet..

[85] Maxwell J.J., Lyerly J.H., Cowger C., Marshall D., Brown-Guedira G., Murphy J.P. (2009). *MlAG12*: A *Triticum timopheevii*-derived powdery mildew resistance gene in common wheat on chromosome 7AL. Theor. Appl. Genet..

[86] Han J., Zhang L., Li G., Zhang H., Xie C., Yang Z., Sun Q.X., Liu Z. (2009). Molecular mapping of powdery mildew resistance gene *MlWE18* in wheat originated from wild emmer *Triticum turgidum* var. *Dicoccoid*. Acta Agronom. Sin..

[87] Ben-David R., Xie W., Peleg Z., Saranga Y., Dinoor A., Fahima T. (2010). Identification and mapping of *PmG16*, a powdery mildew resistance gene derived from wild emmer wheat. Theor. Appl. Genet..

[88] Chhuneja P., Kumar K., Stirnweis D., Hurni S., Keller B., Dhaliwal H.S., Singh K. (2012). Identification and mapping of two powdery mildew resistance genes in *Triticum boeoticum* L. Theor. Appl. Genet..

[89] Tan C., Li G., Cowger C., Carver B.F., Xu X. (2018). Characterization of *Pm59*, a novel powdery mildew resistance gene in Afghanistan wheat landrace PI *Theor*. Appl. Genet..

[90] Xiao M., Song F., Jiao J., Wang X., Xu H., Li H. (2013). Identification of the gene *Pm47* on chromosome 7BS conferring resistance to powdery mildew in the chinese wheat landrace Hongyanglazi. Theor. Appl. Genet..

[91] Ma Q., Luo P.G., Ren Z.L., Huare J., Zujun Y. (2010). Genetic analysis and chromosomal location of two new genes for resistance to powdery mildew in wheat *Triticum aestivum* L. Acta Agron. Sin..

[92] Zhong S., Ma L., Fatima S.A., Yang J., Chen W., Liu T. (2016). Collinearity analysis and high-density genetic mapping of the wheat powdery mildew resistance gene *Pm40* in PI. PLoS ONE.

[93] Dangl J.L., Horvath D.M., Staskawicz B.J. (2013). Pivoting the plant immune system from dissection to deployment. Science.

[94] Marone D., Russo M.A., Laidò G., De Leonardis A.M., Mastrangelo A.M. (2013). Plant Nucleotide Binding Site–Leucine-Rich Repeat (NBS-LRR) genes: Active guardians in host defense responses. Int. J. Mol. Sci..

[95] Dodds P.P., Lawrence G.J., Pryor A., Ellis J.G. (2018). Genetic Analysis and Evolution of Plant Disease Resistance Genes. Ann. Plant Rev. Online.

[96] Keller M., Keller B., Schachermayr G., Winzeler M., Schmid J.E., Stamp P., Messmer M.M. (1999). Quantitative trait loci for resistance against powdery mildew in a segregating wheat x spelt population. Theor. Appl. Genet..

[97] Miedaner T., Flath K. (2007). effectiveness and environmental stability of quantitative powdery mildew (*Blumeria graminis*) resistance among winter wheat cultivars. Plant Breed..

[98] Niks R.E., Qi X.Q., Marcel T.C. (2015). Quantitative resistance to biotrophic filamentous plant pathogens: Concepts, misconceptions and mechanisms. Ann. Rev. Phytopathol..

[99] Golzar H., Shankar M., D’Antuono M. (2016). Responses of commercial wheat varieties and differential lines to western australian powdery mildew (*Blumeria graminis* f. sp. *tritici)* populations. Aust. Plant Pathol..

[100] Cowger C., Mehra L., Arellano C., Meyers E., Paul Murphy J. (2018). Virulence differences in *Blumeria graminis* f. sp. *tritici* from the Central and Eastern United States. Phytopathology.

[101] Shaner G. (1973). Evaluation of slow-mildewing resistance of knox wheat in the field. Phytopathology.

[102] Li Z., Lan C., He Z., Singh R.P., Rosewarne G.M., Chen X., Xia X. (2014). Overview and application of QTL for adult plant resistance to leaf rust and powdery mildew in wheat. Crop Sci..

[103] Pilet-Nayel M.L., Moury B., Caffier V., Montarry J., Kerlan M.C., Fournet S., Durel C.E., Delourme R. (2017). Quantitative resistance to plant pathogens in pyramiding strategies for durable crop protection. Front. Plant Sci..

[104] Aoun M., Breiland M., Turner K., Loladze A., Chao S. (2016). Genome-wide association mapping of leaf rust response in a durum wheat worldwide germplasm collection. Plant Genom..

[105] Liu N., Bai G., Lin M. (2017). Genome-wide association analysis of powdery mildew resistance in U.S. winter wheat. Sci. Rep..

[106] Zhao H.H., Fernando R.L., Dekkers J.C.M. (2007). Power and precision of alternate methods for linkage disequilibrium mapping of quantitative trait loci. Genetics.

[107] Hayes B., Condro C., van der Werf J., Hayes B. (2013). Overview of statistical methods for genome-wide association studies (GWAS). Genome-Wide Association Studies and Genomic Prediction.

[108] Benjamini Y., Hochberg Y. (1995). Controlling the false discovery rate: A practical and powerful approach to multiple testing. J. R Stat. Soc. Ser. B..

[109] Zhang D., Bowden R.L., Yu J., Carver B.F., Bai G. (2014). Association analysis of stem rust resistance in US winter wheat. PLoS ONE.

[110] Liu W., Maccaferri M., Chen X., Laghetti G., Pignone D. (2017). Genome-wide association mapping reveals a rich genetic architecture of stripe rust resistance loci in emmer wheat (*Triticum turgidum* ssp. *dicoccum*). Theor. Appl. Genet..

[111] Li G., Xu X., Tan C., Carver B.F., Bai G., Wang X., Bonman J.M., Wu Y., Hunger R., Cowger C. (2019). Identification of powdery mildew resistance loci in wheat by integrating Genome-Wide Association Study (GWAS) and linkage mapping. Crop J..

[112] Moseman J.G., Nevo E., Morshidy M.A.E., Zohary D. (1984). Resistance of *Triticum dicoccoides* to infection with *Erysiphe graminis tritici*. Euphytica.

[113] Xie W., Nevo E. (2008). Wild emmer: Genetic resources, gene mapping and potential for wheat improvement. Euphytica.

[114] Huang L., Raats D., Sela H., Klymiuk V., Lidzbarsky G., Feng L. (2016). Evolution and adaptation of wild emmer wheat populations to biotic and abiotic stresses. Ann. Rev. Phytopathol..

[115] Laidò G., Mangini G., Taranto F., Gadaleta A., Blanco A., Cattivelli L., Marone D., Mastrangelo A.M., Papa R., De Vita P. (2013). Genetic diversity and population structure of tetraploid wheats (*Triticum turgidum* L.) estimated by SSR, DArT and pedigree data. PLoS ONE.

[116] Juliana P., Singh R.P., Singh P.K., Poland J.A., Bergostrom G.C. (2018). Genome-wide association mapping for resistance to leaf rust, stripe rust and tan spot in wheat reveals potential candidate genes. Theor. Appl. Genet..

[117] EnsemblPlants. https://plants.ensembl.org/index.html.

[118] Peterson R.F., Campbell A.B., Hannah A.E. (1948). A diagrammatic scale for rust intensity on leaves and stems of cereals. Can. J. Res..

[119] Piarulli L., Gadaleta A., Mangini G., Signorile M.A., Pasquini M., Blanco A., Simeone R. (2012). Molecular identification of a new powdery mildew resistance gene on chromosome 2BS from *Triticum turgidum* ssp. *dicoccum*. Plant Sci..

[120] Dellaporta S.L., Wood J., Hicks J.B. (1983). Isolation of DNA from higher plants. Plant Mol. Biol. Rep..

[121] TraitGenetics. http://www.traitgenetics.de.

[122] Marcotuli I., Houston K., Schwerdt J.G., Waugh R., Fincher G.B., Burton R.A., Blanco A., Gadaleta A. (2016). Genetic diversity and genome wide association study of β-glucan content in tetraploid wheat grains. PLoS ONE.

[123] Buckler Lab. http://www.maizegenetics.net..

[124] Zhang Z.W., Ersoz E., Lai C.Q., Todhunter R.J., Tiwari H.K. (2010). Mixed linear model approach adapted for genome-wide association studies. Nat. Genet..

