# Supplementary material for: Mapping Powdery Mildew (Blumeria graminis f. sp. tritici) Resistance in Wild and Cultivated Tetraploid Wheats
Source: Int J Mol Sci. 2020 Oct 24;21(21):7910. doi: 10.3390/ijms21217910 (PMC7662567; doi:10.3390/ijms21217910)
Supplement: Supplementary file 1 [file ijms-21-07910-s001.zip › Supplemental Materials/Figure S1.docx]

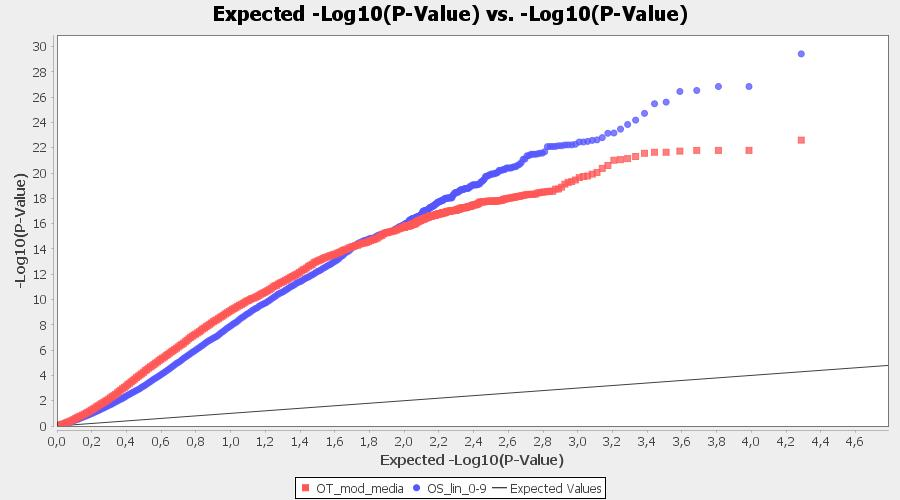

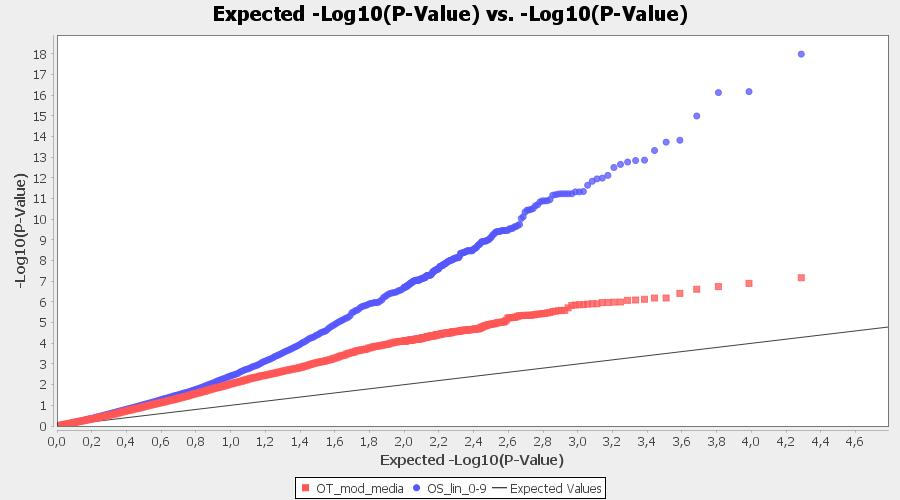


1. **b)**


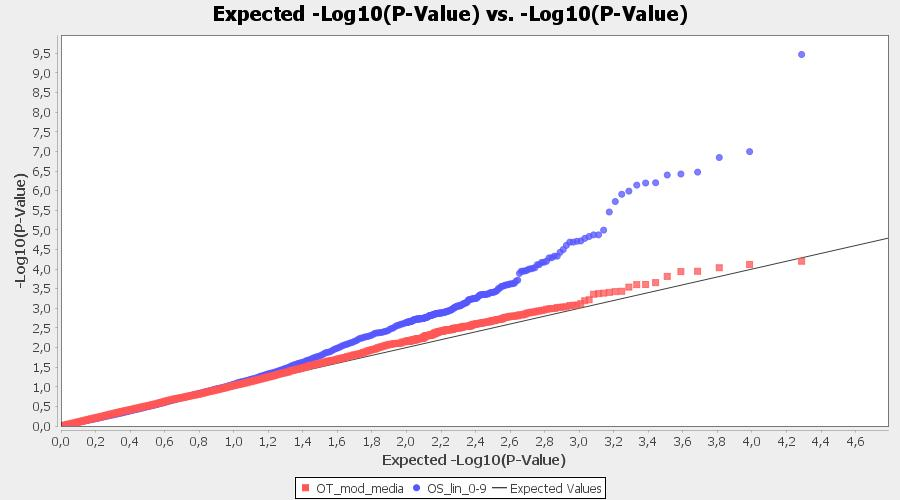

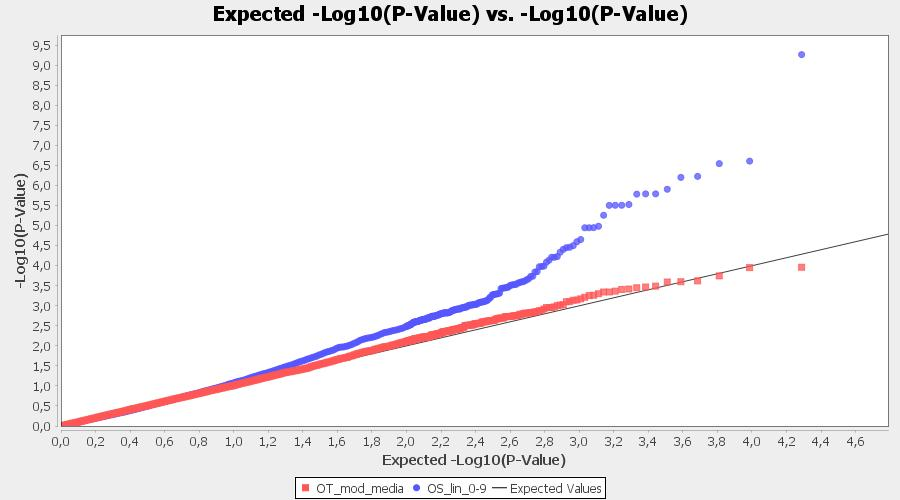


**c) d)**

**Figure S1.** Genome-wide association analysis for adult plant resistance and seedling resistance in a tetraploid wheat collection. Quantile-quantile (Q-Q) plots of the observed –log10 (P) values (y axes) against the expected distribution of –log10 (P) values (x axes) for the models: a) GLM , b) GLM +Q, c) MLM+K, d) MLM+K+Q. Red line = Adult plants. Blue line = seedlings
